# Effects of Valproic Acid Supply Shortage on Pharmacy Operations in a Region of Japan

**DOI:** 10.7759/cureus.65324

**Published:** 2024-07-25

**Authors:** Satoru Matsunuma, Shigeki Sunaga, Kanami Hirose, Gaku Samizo, Ryohei Soeishi, Koichi Yoshimoto, Hiroyuki Jimbo

**Affiliations:** 1 Department of Pharmacy, Tokyo Medical University Hachioji Medical Center, Tokyo, JPN; 2 Department of Neurosurgery, Tokyo Medical University Hachioji Medical Center, Tokyo, JPN; 3 Division of Pharmacy, Hachioji Pharmaceutical Center, Tokyo, JPN

**Keywords:** survey, questionnaire, pharmacy, drug supply shortage, valproic acid

## Abstract

Introduction

Valproic Acid (VPA) is an essential drug in epilepsy treatment, yet it has faced supply instability in Japan. The extent of the VPA shortage and the associated increase in pharmacists' workload and collaboration with healthcare organizations remains unclear. This study investigates the potential effects of these disruptions on the roles of pharmacists.

Methods

A questionnaire was administered to pharmacies in Hachioji City, Japan. The survey addressed inventory management, patient complaints, and the potential effects on pharmacy operations during VPA supply instability. A chi-squared test of independence was conducted to compare the most unstable VPA supply period with the current supply situation. Supply stability according to pharmacy characteristics such as the number of prescriptions received per day and primary patient age group was also evaluated.

Results

Of the 42 pharmacies surveyed, 76.2% reported changes in prescription processing due to VPA supply issues. The main challenges were increased workload in inventory management and patient concerns regarding medication availability and quality. Pharmacies primarily serving clinical prescriptions and pediatric patients were the most affected by the supply instability.

Discussion

This study highlighted the potential effects of VPA supply instability on pharmacy operations. Pharmacists are expected to provide continuous treatment to patients through effective counseling and medication guidance to alleviate anxiety and concerns related to supply shortages.

## Introduction

Valproic acid (VPA) possesses high efficacy and safety for generalized and unclassified epilepsy and is an essential antiseizure medication (ASM) in Japan [1]. A network meta-analysis concluded that VPA has the best profile compared to all other ASMs and is the first-choice drug for generalized epilepsy per the guidelines of the Japanese Society of Neurology [2,3]. According to calculations using the Defined Daily Dose by the World Health Organization and prescription amounts obtained from the National Database of Health Insurance Claims and Specific Health Checkups Open Data Japan, VPA had the largest proportion of ASM prescriptions in Japan for the fiscal year 2020 [4,5].

Following administrative actions against two generic drug manufacturers in Japan for legal violations, the supply of VPA decreased, leading to unstable supply nationwide [6]. Since August 2021, various pharmaceutical companies began halting and adjusting VPA shipments, and in October 2021, the Japan Epilepsy Society issued recommendations concerning the instability of VPA supply. Subsequently, the supply situation gradually improved. By October 2023, all shipment adjustments for VPA had been lifted. Although lamotrigine and levetiracetam are recommended as alternative monotherapy drugs for generalized epilepsy to replace VPA, the choice of ASMs depends on the individual patient profiles, and not all patients experience unchanged seizure frequency when their medication is switched [2]. Seizures can lead to serious consequences such as injury or death to a patient or others, and the loss of a driving license. Therefore, pharmacists can bear a large burden in ensuring VPA is stocked and adjusting prescription volumes compared to other medications. The aforementioned guidelines recommend not switching from brand-name to generic drugs when seizure management is under control [3]. However, in situations where certain medications are unavailable, changes have been unavoidable due to stock situations. Psychological changes in patients due to medication adjustments can affect the control of seizures [7], which underscores the importance of pharmacist medication guidance. From the perspective of providing medication guidance, the burden on pharmacists is likely to have increased.

A questionnaire survey conducted by the Japan Epilepsy Society in 2022 revealed that 28% of physicians chose ASMs other than VPA when starting new treatments, and 6% attempted to discontinue or change VPA administration in existing therapies [8]. Furthermore, 55% of the respondents indicated that they were aware of this supply instability. However, the unavailable portion of the initial VPA supply remains uncertain. Additionally, the extent to which the decreased VPA supply has heightened the burden on pharmacists and the adequacy of collaboration with medical institutions to address this issue are both unclear.

This study investigated the effects of an unstable VPA supply on the roles of pharmacists in local pharmacies in Japan using a questionnaire survey.

## Materials and methods

Study subjects and period

We surveyed member pharmacists of the Hachioji Pharmacists Association in Hachioji City, Tokyo, Japan, to investigate the impact of VPA supply instability on their work. Pharmacies that had not received prescriptions for VPA for epilepsy treatment since January 2020 were excluded to remove pharmacies that had not handled VPA prescriptions since before the VPA shipment adjustment. The survey period was from July 10, 2023 to August 25, 2023.

Questionnaire development

The questionnaire was developed based on the guidelines for questionnaire surveys [9]. This involved generating, reducing, and revising question items, followed by a pre test. Responses were primarily binary options or Likert scales. The questionnaire is written in Japanese and includes sections on the target respondents, time required to complete the questionnaire, implementation period for the questionnaire, study overview, ethical considerations, and question statements. The questions mainly focus on pharmacy characteristics, current and past VPA supply situations, patient complaints, the impact on pharmacists’ work, and recommendations for medication adjustments.

Questionnaire testing

Referring to the previously mentioned guidelines, pre-tests were conducted [9]. To test the questionnaire’s comprehensiveness and clarity, we administered the questionnaire in person to two pharmacists from the study group. Subsequently, we assessed the questionnaire’s clinical sensibility (ability to discriminate among responses, face validity, content validity, and ease of use) and specificity tables (to determine the relevance and appropriateness of the questions in relation to collected items and research participants) using a 4-point Likert scale by administering it to five pharmacists from the Hachioji Pharmacists Association excluding study group members. The questionnaire was revised and finalized based on the results of the pre tests. Supplementary Material S1 (see Appendices) presents the content of the questionnaire. An English-translated version of the questionnaire is also available as Supplementary Material S2 (see Appendices).

Questionnaire administration

Google Forms (Google LLC, Mountain View, CA, USA) was used to collect and compile the responses. The Hachioji Pharmacists Association sent respondents e-mails containing the link to the questionnaire. If more than one pharmacist at a given pharmacy was a member of the Hachioji Pharmacists Association, they were asked to combine their answers; therefore, one questionnaire per pharmacy was provided. If multiple responses were received from the same pharmacy, the most recent one was used. A reminder e-mail was sent during the implementation period to reduce non-response errors. Only the pharmacy name and e-mail address were collected for personal information to avoid duplicate responses. The collected information was stored on an external hard drive with a password and maintained in a locked cabinet.

Statistical analyses

A chi-squared test of independence was conducted to compare the most unstable VPA supply period with the current supply situation. The number of prescriptions handled daily by each pharmacy was categorized into two groups using the median as the cut-off value. The chi-squared test was also used to assess differences in supply stability based on the number of prescriptions received daily, the primary source of prescriptions (clinic or hospital), and the primary patient age group (adult or pediatric). Unanswered responses were excluded from the analyses. Statistical analyses were performed using SPSS version 29 (IBM, Armonk, NY, USA). The level of statistical significance was set at 5%. The sample size was calculated based on the assumption of detecting a 30% absolute change in the proportion of prescription changes experienced, shifting from 50% in the control group to 20% in the treatment group. This calculation considered a standard deviation of ±5, aiming to achieve a study power of 80% and a significance level of 5%. The sample size was calculated to be 78 based on these conditions.

Ethical approval and participant consent statement

This study was performed in accordance with the Declaration of Helsinki and was approved by the Tokyo Medical University Ethics Committee (Approval No. T2023-0034). Participation in the survey was voluntary, and consent was obtained from the respondents before they completed the questionnaire.

## Results

Characteristics of responding pharmacies

The survey was submitted to 162 pharmacies of the Hachioji Pharmacists Association and responses were received from only 65 facilities (response rate: 40.1%). Of these 65 facilities, 23 had not dispensed VPA for epilepsy treatment since January 1, 2020; hence, they were excluded. The remaining 42 facilities (64.6%: 42/65) were included in the analyses. The median number of prescriptions handled daily by the included pharmacies was 70, with 52.4% (n=22) handling fewer than 70 prescriptions and 47.6% (n=20) handling 70 or more. The primary sources of VPA prescriptions were clinics (54.8%, n=23) and pharmacies (38.1%, n=16); 7.1% (n=3) were unspecified. The primary patient age groups were adult-only (64.3%, n=27), pediatric-only (0%), and adult and pediatric patients (35.7%, n=15).

Pharmacist responses and anxiety levels during supply instability

**Figure 1:**
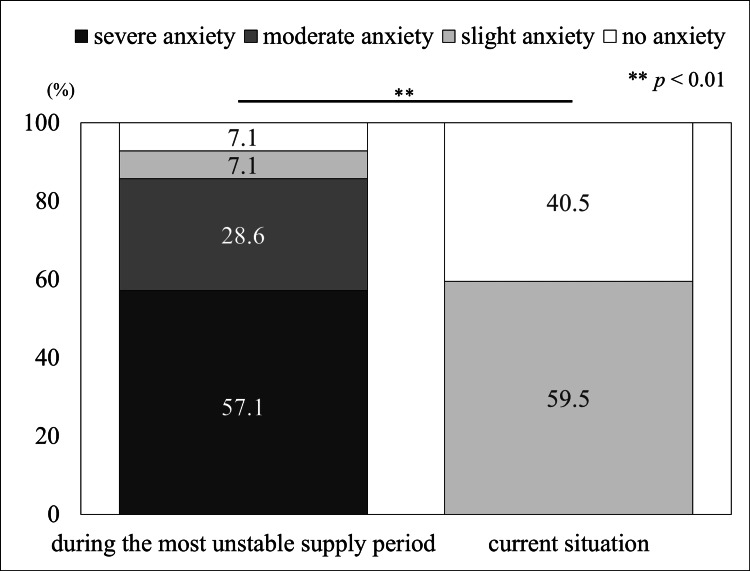
Pharmacist reactions to valproic acid supply instability. Statistical analysis was performed using the chi-squared test.

The responses regarding supply instability at each pharmacy are shown in Figure 1.

During the most unstable period, 57.1% (n=24) of pharmacists reported “severe anxiety,” whereas 28.6% (n=12) reported “moderate anxiety.” In the current situation, there were no reports of both “severe anxiety” and “moderate anxiety,” and this change in response was statistically significant (*p* < 0.01). The reactions, categorized by the daily number of prescriptions received, main source of prescriptions, and primary patient age groups, are presented in Table 1.

**Table 1:** Pharmacy characteristics and pharmacists’ reactions to the valproic acid supply instability. Statistical analysis was performed using the chi-squared test.

During the most unstable supply period		Severe anxiety % (n)	Moderate anxiety % (n)	Slight anxiety % (n)	No anxiety % (n)	*p*-value
Number of prescriptions handled daily	< 70	50 (11)	31.8 (7)	13.6 (3)	4.5 (1)	0.290
	≥ 70	65 (13)	25 (5)	0	10 (2)	
Primary sources of valproic acid prescriptions	clinic	69.6 (16)	26.1 (6)	0	4.3 (1)	0.221
	hospital	43.8 (7)	37.5 (6)	12.5 (2)	6.2 (1)	
Primary patient age groups	adults	40 (14)	45.7 (16)	8.6 (3)	5.7 (2)	0.557
	adults and pediatrics	66.7 (10)	26.7 (4)	0	6.6 (1)	
Current situation		Severe anxiety % (n)	Moderate anxiety % (n)	Slight anxiety % (n)	No anxiety % (n)	*p*-value
Number of prescriptions handled daily	< 70	0	0	63.6 (14)	36.4 (8)	0.569
	≥ 70	0	0	55 (11)	45 (9)	
Primary sources of valproic acid prescriptions	clinic	0	0	73.9 (17)	26.1 (6)	0.023
	hospital	0	0	37.5 (6)	62.5 (10)	
Primary patient age groups	adults	0	0	48.1 (13)	51.9 (14)	0.044
	adults and pediatrics	0	0	80 (12)	20 (3)	

There was no significant difference in the responses during the most unstable period across the groups. In the current situation, for pharmacies whose primary source of prescriptions were clinics, 73.9% (n=17) and 26.1% (n=6) reported “slight anxiety” and “no anxiety,” respectively; for those in which hospitals were the primary source, 37.5% (n=6) and 62.5% (n=10) reported “slight anxiety” and “no anxiety,” respectively (*p* = 0.023). For pharmacies primarily attending adult patients, 48.1% (n=13) and 51.9% (n=14) indicated experiencing “slight anxiety” and “no anxiety,” respectively, and 80% (n=12) and 20% (n=3) of those dealing with both adult and pediatric patients reported “slight anxiety” and “no anxiety,” respectively (*p* = 0.044).

**Figure 2:**
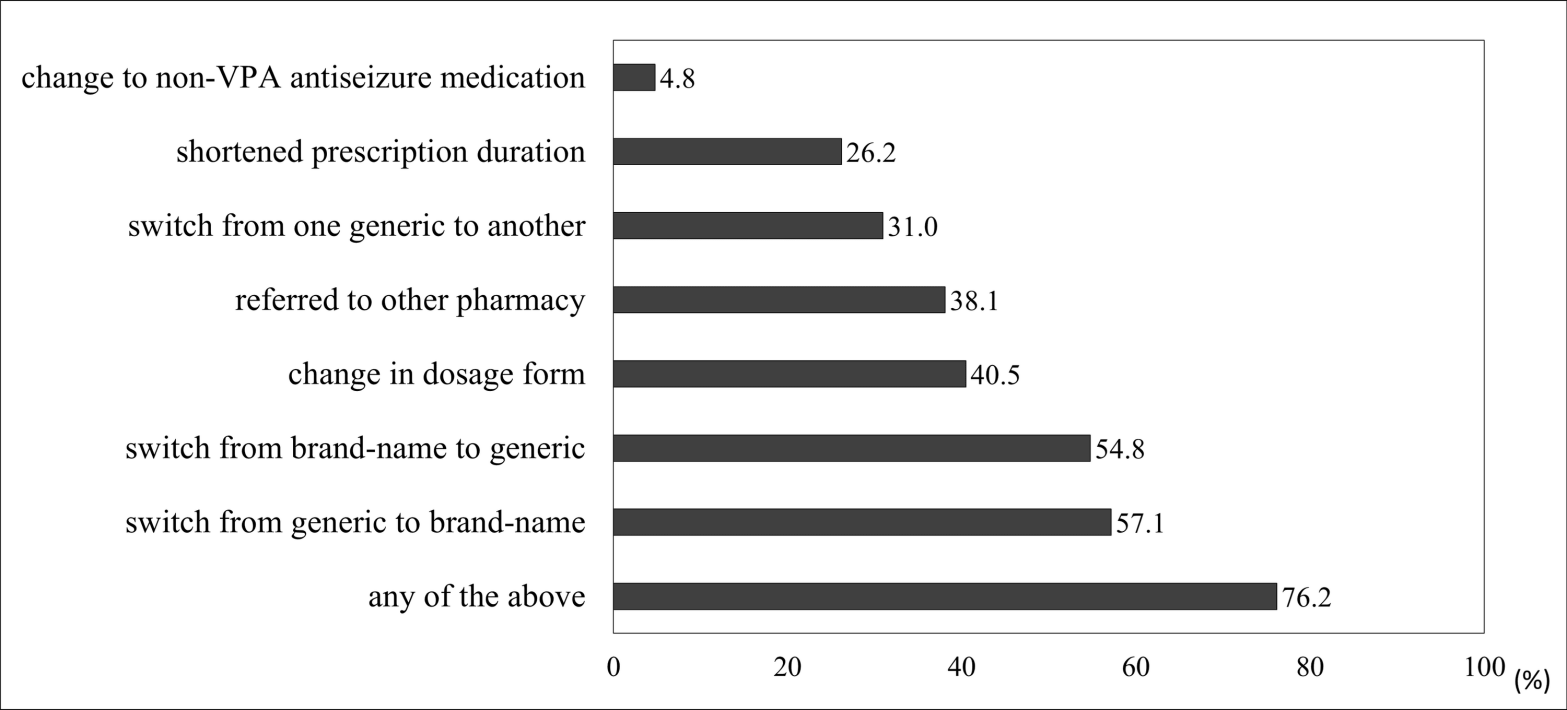
Adjustments to prescription filling due to valproic acid supply instability. VPA, valproic acid.

Regarding adjustments made in response to VPA supply instability, 76.2% (n=32) of pharmacists performed at least one form of modification during prescription processing (Fig. 2).

The most common modification was switching from generic to brand-name drugs (57.1%, n=24), followed by switching from brand-name to generic drugs (54.8%, n=23). Some pharmacies referred patients to other pharmacies (38.1%, n=16). Changing to non-VPA ASMs was the least common adjustment (4.8%, n=2). The responses, categorized by the daily number of prescriptions received, main source of prescriptions, and primary patient age groups, are presented in table 2.

**Table 2:** Adjustments to prescription filling based on pharmacy characteristics. Statistical analysis was performed using the chi-squared test.

		Experience of valproic acid prescription changes		
		Yes % (n)	No % (n)	*p*-value
Number of all prescriptions handled daily	< 70	68.2 (15)	31.8 (7)	0.201
	≥ 70	85 (17)	15 (3)	
Primary sources of valproic acid prescriptions	clinic	82.6 (19)	17.4 (4)	0.312
	hospital	68.8 (11)	31.2 (5)	
Primary patient age groups	adults	66.7 (18)	33.3 (9)	0.052
	adults and pediatrics	93.3 (14)	6.7 (1)	

The rate of prescription modifications was higher in pharmacies serving both adult and pediatric patients than in those serving only adults, although the difference was not significant (93.3% vs. 66.7%, *p* = 0.052). Similarly, no significant variation was observed in the rates of prescription adjustments based on primary sources of prescriptions or number of prescriptions received daily.

Patient complaints and pharmacy adjustments

**Figure 3:**
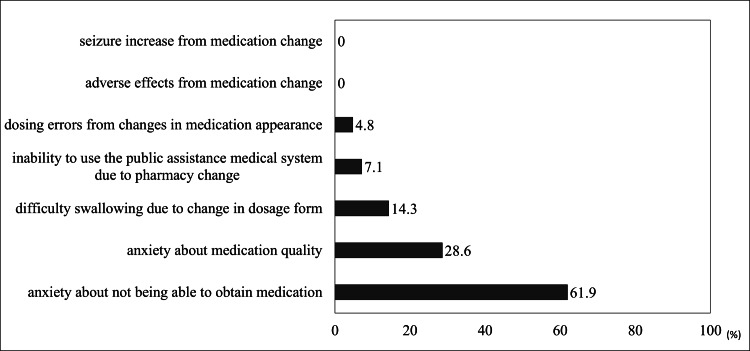
Patient complaints associated with valproic acid supply instability.

Patient complaints received by pharmacists are shown in Figure 3.

Responses were collected if the pharmacy had encountered the situation at least once. The frequent issue reported was anxiety about not being able to obtain medication (61.9%, n=26) and anxiety about medication quality (28.6%, n=12). There were no reports of increased epileptic seizures or emergence or worsening of side effects due to medication changes.

Workload of pharmacists and cooperation with other health care providers

**Figure 4:**
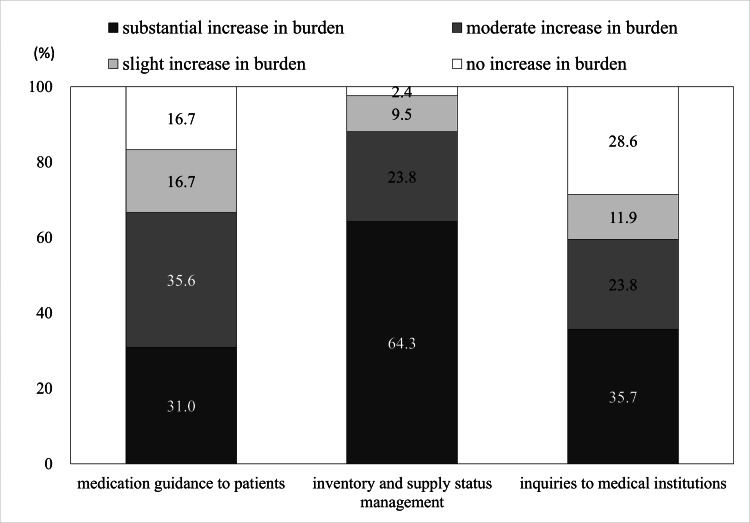
Pharmacist workload burdens due to valproic acid supply instability.

Questions on the impact of VPA supply instability on pharmacists’ roles consisted of three items: “medication guidance to patients,” “inventory and supply status management,” and “inquiries to medical institutions” (Fig. 4).

The greatest increase in workload was reported in “inventory and supply status management,” with 64.3% (n=27) of pharmacists indicating a “substantial increase in burden” and 23.8% (n=10) reporting a “moderate increase in burden.”

**Figure 5:**
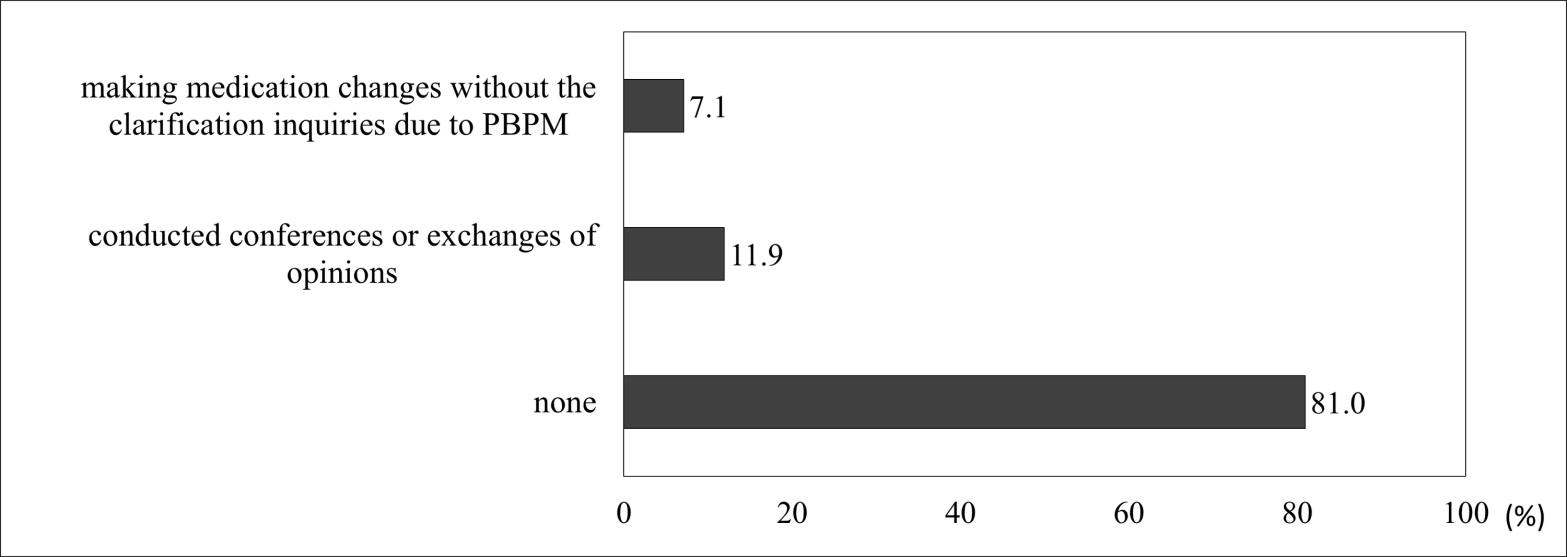
Pharmacist collaborations with physicians and medical institutions due to valproic acid supply instability. PBPM, protocol-based pharmaceutical management.

The results regarding the collaboration of pharmacists with physicians and medical institutions are presented in Figure 5.

Only 7.1% (n=3) of pharmacists reported making medication changes using protocol-based pharmaceutical management (PBPM) without the need for clarification inquiries, and merely 11.9% (n=5) conducted conferences or exchanged opinions.

Status of recommendation to switch medication

**Figure 6:**
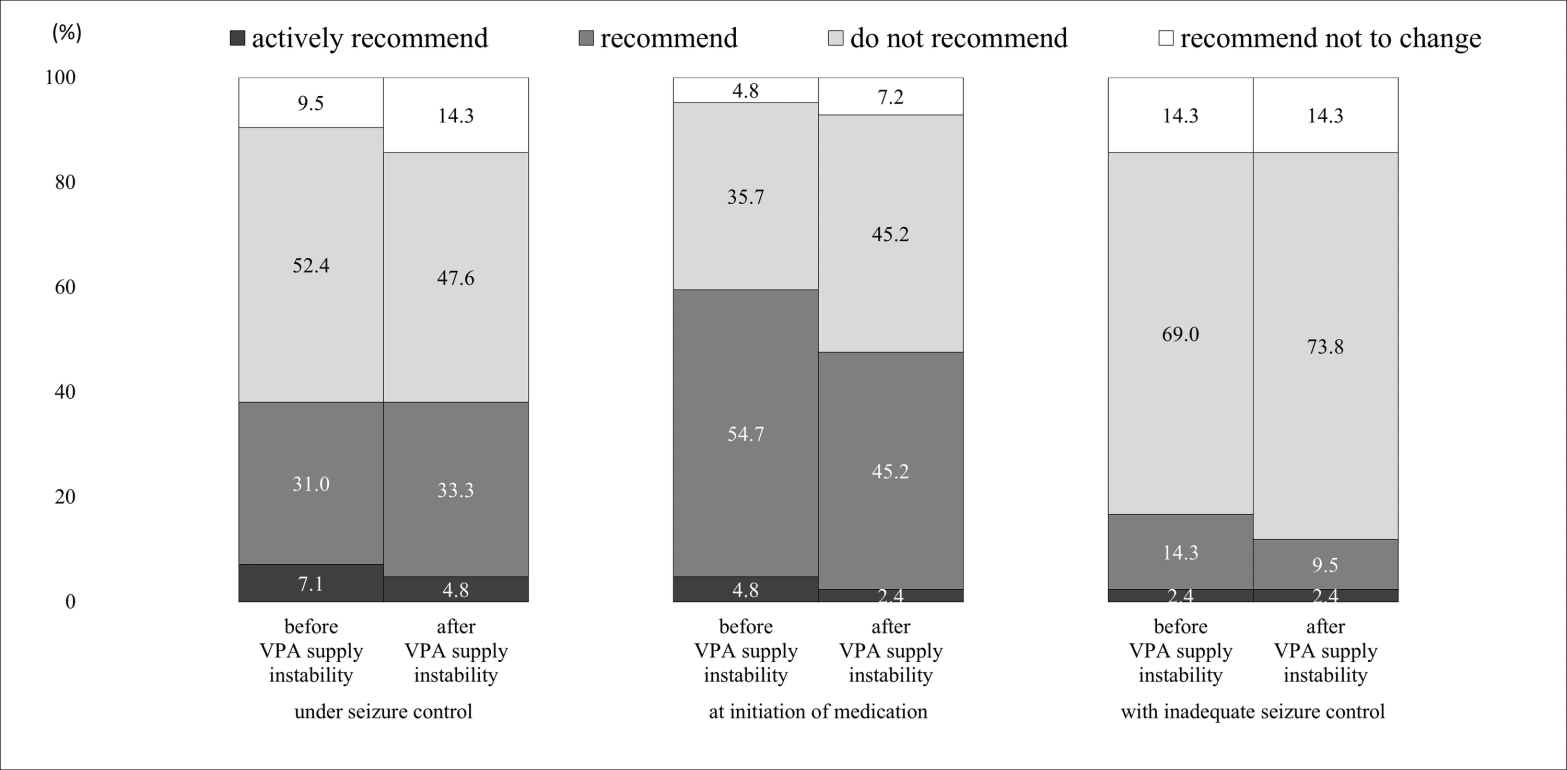
Pharmacist recommendations to patients regarding switching to generic alternative antiseizure medicines. VPA, valproic acid.

Figure 6 shows pharmacist recommendations to patients regarding switching to generic ASMs before and after supply instability.

According to the epilepsy clinical guidelines in Japan, "It is recommended not to switch to generics in patients whose seizures are well-controlled," thus responses were obtained based on the seizure control status of the patients [3]. There was no notable change in the recommendations for generics before and after VPA supply instability for all patients. Approximately 40% of pharmacists reported that they recommended a switch to generic ASMs for patients under seizure control during the two periods.

## Discussion

This study was the first to investigate in detail the impact of the unstable VPA supply in Japan on community pharmacies and clarified the specific effects on pharmacist workloads and patient care.

The study results revealed that the unstable VPA supply had a serious impact on pharmacy operations. Notably, pharmacists experienced a significant increase in workload related to inventory and supply management. According to the survey, 76.2% (n=32) of respondents were forced to request prescription changes from their physicians due to supply shortages. Pharmacies that primarily handled prescriptions from clinics and those serving pediatric patients were particularly affected. Pharmaceutical wholesalers often supply medicines according to the purchasing history of pharmacies. Therefore, pharmacies that receive prescriptions from clinics may have been affected more by the supply instability because they have a smaller supply than pharmacies servicing hospitals due to the smaller number of patients they serve. The high prevalence of generalized epilepsy and the frequent use of VPA as a first-line drug in pediatric epilepsy may explain why pharmacies dealing with pediatric patients were particularly affected [10,11].

Regarding the complaints pharmacists received from patients, "anxiety about not being able to obtain medication" and "anxiety about medication quality" were the most common. These complaints suggest that they may have impacted patients’ psychological health. Anxiety is known to be associated with worsening epilepsy and reduced quality of life [7,12]. A survey of patients with epilepsy in Germany found that patients with supply difficulties used treatments with more ASMs and had higher adverse event scores [13]. Medication guidance and counselling by pharmacists may improve patients' quality of life regarding their anxieties [14]. In situations of unstable ASM supply, counseling patients about medication changes and follow-up after changes are crucial.

According to existing literature, medication shortages have a severe impact on healthcare settings and patient safety [15-18]. Medication shortages force ingredient changes and switch to generics, which are highly stressful for all stakeholders, including pharmacists, doctors, pharmaceutical wholesalers, nurses, and patients [19,20]. The unstable supply of many medicines, not just VPA, in Japan, is probably increasing the burden of pharmacist inventory management. Changing the class of ASMs may cause seizure recurrence if the drugs control epileptic seizures. Furthermore, worsening of clinical conditions has been reported in several cases due to switches to generic ASMs; the pros and cons of switching to generics are debated [21-23]. In the aforementioned survey, a switch to a generic drug occurred in 39.4% of patients affected by supply difficulties [13]. Seizure occurrence can lead to significant events such as life-threatening situations. Therefore, pharmacists may be more burdened in responding to supply instability for ASMs than for other drugs.

The expansion of the generic drug market and intensified price competition have made economic sustainability particularly challenging for small pharmaceutical companies, resulting in unstable supplies [24]. One of the reasons behind these incidents is the Japanese government's policies to reduce medical costs to protect the country's universal health coverage, which is in a state of crisis due to the super-aging society. Aggressive policies promoting generic drugs hinder quality assurance and the improvement of domestic regulations, thereby reducing supply reliability. These impacts extend to medications such as ASMs, which are difficult to substitute. According to a study that analyzed the Australian drug shortage reporting database, 93% of the ASM supply shortages were for generic drugs [25]. Lowering drug prices, including those of generics, contributes to reducing national healthcare expenditures. However, in addition to the increased burden and labor costs for medical staff due to unstable supply and patient anxiety, the cost of medical care in the event of seizure recurrence must also be considered. Medical costs for the treatment of recurrent epileptic seizures and the transition to status epilepticus superimposed are estimated at 4 billion USD per year in the United States of America and over 83 million EUR in Germany [26,27]. To avoid causing these unnecessary increases in healthcare costs, strategies need to be developed to mitigate drug shortages, avoid medication misallocation, and ensure the long-term quality and security of medicines [28].

Limitations

This study had several limitations. The questionnaire survey was limited to a specific region in Japan, and the findings may not be applicable to other regions. The perception of anxiety is subjective, and this may have led to potential bias. Since it was unclear when the period of greatest VPA supply instability occurred, it could not be predefined. The determination of the most unstable period was dependent on respondents' subjective judgment, which may have introduced bias into the research findings. The changes in response rates due to various factors did not align with the calculated sample size. Patient complaints are not reliable indicators of the status epileptic or adverse effects. We were unable to obtain information regarding the mental health and well-being of pharmacists, and the impact on their work motivation remains a subject for future investigation. Despite these limitations, this was the first study to investigate the effect of VPA supply instability on pharmacies.

## Conclusions

This survey has clarified the impact of the unstable VPA supply on pharmacists in specific regions of Japan. Pharmacies dispensing prescriptions from clinics and those dispensing for pediatric patients struggled to secure VPA and found stock management tasks to be a significant burden. Policymakers are expected to ensure a stable supply of essential medications, including VPA, even if additional costs must be accepted. Any change in medication requires close follow-up, especially monitoring of epileptic seizures and adverse effects after a change. Pharmacists are expected to provide adequate counselling and medication guidance to reduce patient anxiety and provide ongoing treatment.

## Appendices

Supplemental material S1:  Content of the questionnaire (Japanese)

対象　　：八王子薬剤師会所属薬局の管理薬剤師

所要時間：10-15分

実施期間：2023年7月10日(月) から2023年8月25日(金) まで

概要　　：バルプロ酸製剤の供給不安定における実態調査

倫理的配慮：本研究は東京医科大学医学倫理委員会で承認されています（承認番号：T2023-0034）。アンケートへの回答をもって本研究への参加の同意とさせていただきます。

大変お忙しい中恐縮ですが、ご協力のほどよろしくお願い致します。

1. 2020年1月1日より現在までにてんかん治療のためのバルプロ酸製剤を調剤する機会がありましたか。

はい

いいえ

Branching Instructions

IF ANSWER TO (QUESTION# 1 is (Yes)) THEN GO TO QUESTION# 2 IF ANSWER TO (QUESTION# 1 is (No)) THEN STOP, YOU HAVE FINISHED THIS SURVEY.
 

2. 2020年1月時点での、1日に応需する処方箋の平均枚数を教えてください。

3. バルプロ酸製剤の主な処方箋発行元の許可病床数を教えてください。（診療所・クリニックの場合は0とご回答ください）

4. バルプロ酸製剤を調剤する患者さんは小児または成人のどちらですか。（各年間1名以上であれば「成人および小児」とご回答ください）

成人のみ

小児のみ（15歳未満）

成人および小児

5. 最も流通が不安定であった際の需要に対するバルプロ酸製剤の供給の状況を教えてください。

問題なし

少し不安があった

不安があった

とても不安があった

6. 現在の需要に対するバルプロ酸製剤の供給の状況を教えてください。

問題なし

少し不安がある

不安がある

とても不安がある

7. 供給不安定を理由とした、バルプロ酸製剤の処方変更依頼等の経験を教えてください。

7-1. 処方日数を短くしてもらった （または短い日数分を先に調剤し、不足分を後日調剤した）

ある

ない

7-2. 別のバルプロ酸製剤に剤形の変更

ある

ない

7-3. 後発品から先発品へ変更

ある

ない

7-4. 先発品から後発品へ変更

ある

ない

7-5. 後発品から後発品（他のメーカー）へ変更

ある

ない

7-6. 散剤がなく、錠剤を粉砕

ある

ない

7-7 バルプロ酸製剤以外の製剤への変更

ある

ない

7-8 別の薬局に行ってもらった

ある

ない

7-9 その他（自由回答）

8. バルプロ酸製剤の供給不安定による、薬剤師業務への影響について教えてください。

8-1. 患者さんへの説明・服薬指導

大いに負担が増加した

負担が増加した

少し負担が増加した

変わらない

8-2. 在庫管理業務（卸や製薬会社への供給状況確認等も含む）

大いに負担が増加した

負担が増加した

少し負担が増加した

変わらない

8-3. 医療機関への疑義照会・問い合わせ・連絡

大いに負担が増加した

負担が増加した

少し負担が増加した

変わらない

9. 患者さん（またはご家族）からバルプロ酸製剤の供給不安定により下記の訴えを受けたことはありますか。

9-1. 薬が変更になったことでてんかん発作が増えた

ある

ない

9-2. 薬が変更になったことで副作用が起こった（または増えた）

ある

ない

9-3. 自分の薬が入手できなくなる不安がある

ある

ない

9-4. 自分が使用している薬の品質ついて不安がある

ある

ない

9-5. 薬剤変更に伴い名前や見た目が変わったことで飲み間違いがあった

ある

ない

9-6. 剤形（錠剤、散剤、水剤）が変わったことで、飲みにくくなった

ある

ない

9-7. 薬局の変更により自立支援医療制度が利用できない

ある

ない

9-8. その他（自由回答）

10. バルプロ酸製剤の供給不安定に対して、患者さんの制限や処方の変更について、医師とのカンファレンスやプロトコール制定を行ったことはありますか（個々の処方に対する疑義照会を除く）

カンファレンスや意見交換を行った

プロトコールに基づく薬物治療管理（厚労省医政局長通知, 医政発0430第1号）を行っており、疑義照会なしで剤形の変更等を行うなどの対応をした

なし

11. 後発医薬品が供給不安定となる状況以前において、後発品への変更について教えてください。 抗てんかん薬以外の薬剤と同様の扱いであれば、その対応でご回答ください。

11-1. 先発医薬品内服下で発作が抑制されている状態の患者さんにおいて、抗てんかん薬を後発品へ変更することをすすめていましたか。

積極的にすすめていた

すすめていた

すすめていなかった

変更しないことをすすめていた

11-2. 新規に抗てんかん薬が開始される患者さんにおいて、抗てんかん薬を後発品へ変更することをすすめていましたか。

積極的にすすめていた

すすめていた

すすめていなかった

変更しないことをすすめていた

11-3. 先発医薬品内服下で発作が抑制されていない状態の患者さんにおいて、抗てんかん薬を後発品へ変更することをすすめていましたか。

積極的にすすめていた

すすめていた

すすめていなかった

変更しないことをすすめていた

12. 後発医薬品が供給不安定となった現在において、後発品への変更について教えてください。 抗てんかん薬以外の薬剤と同様の扱いであれば、その対応でご回答ください。

12-1. 先発医薬品内服下で発作が抑制されている状態の患者さんにおいて、抗てんかん薬を後発品へ変更することをすすめていましたか。

積極的にすすめていた

すすめていた

すすめていなかった

変更しないことをすすめていた

12-2. 新規に抗てんかん薬が開始される患者さんにおいて、抗てんかん薬を後発品へ変更することをすすめていましたか。

積極的にすすめていた

すすめていた

すすめていなかった

変更しないことをすすめていた

12-3. 先発医薬品内服下で発作が抑制されていない状態の患者さんにおいて、抗てんかん薬を後発品へ変更することをすすめていましたか。

積極的にすすめていた

すすめていた

すすめていなかった

変更しないことをすすめていた

13. バルプロ酸製剤を含む抗てんかん薬を後発医薬品へ変更し調剤した際の医療機関への変更報告手段の中で、最も用いる頻度が高いものを教えてください

FAX

電話

お薬手帳への記載

その他（自由記載）

14.　その他、ご意見がございましたらご回答をお願い致します。

Supplemental material S2:  Content of the questionnaire (English translation of the original Japanese text)

Target: Pharmacist managers of pharmacies affiliated with the Hachioji Pharmacists Association

Time required: 10-15 minutes

Implementation period: From Monday, July 10, 2023, to Friday, August 25, 2023

Overview: Survey on the actual conditions of supply instability of valproate preparations

Ethical considerations: This study has been approved by the Medical Ethics Committee of Tokyo Medical University (Approval No.: T2023-0034). Participation in this study is considered consent to participate by responding to the questionnaire.

We apologize for the inconvenience during your busy schedule and appreciate your cooperation.

1. Have you had the opportunity to dispense valproate preparations for the treatment of epilepsy since January 1, 2020, to the present?

Yes

No

Branching Instructions:

IF ANSWER TO QUESTION 1 is "Yes" THEN GO TO QUESTION 2

IF ANSWER TO QUESTION 1 is "No" THEN STOP, YOU HAVE FINISHED THIS SURVEY.

2. Please tell us the average number of prescriptions you handled per day as of January 2020.

3. Please provide the number of authorized beds at the main prescription issuance sources for valproate preparations. (If a clinic, please answer 0.)

4. Are the patients for whom you dispense valproate preparations adults or children? (If at least one person per year is in each category, please answer "Both adults and children.")

Adults only

Children only (under 15 years old)

Both adults and children

5. Please tell us about the supply situation of valproate preparations to meet demand during the most unstable period of supply.

No anxiety

Slight anxiety

Moderate anxiety

Severe anxiety

6. Please tell us about the current supply situation of valproate preparations to meet demand.

No anxiety

Slight anxiety

Moderate anxiety

Severe anxiety

7. Please share your experience of requesting prescription changes due to supply instability of valproate preparations.

7-1. Shortened prescription duration (or dispensed a short period first and the rest later)

Yes

No

7-2. Changed in dosage form

Yes

No

7-3. Switched from generic to brand-name

Yes

No

7-4. Switched from brand-name to generic

Yes

No

7-5. Switched from one generic to another (different manufacturer)

Yes

No

7-6. Crushed tablets when powder form was unavailable

Yes

No

7-7. Changed to non-valproate antiseizure medication

Yes

No

7-8. Referred to other pharmacy

Yes

No

7-9. Other (open-ended)

8. Please tell us about the impact of the supply instability of valproate preparations on pharmacy operations.

8-1. Explanation and guidance to patients

Substantial increase in burden

Slight increase in burden

Moderate increase in burden

No increase in burden

8-2. Inventory management (including checking supply status with wholesalers or pharmaceutical companies)

Substanial increase in burden

Slight increase in burden

Moderate increase in burden

No increase in burden

8-3. Inquiries and communication with medical institutions

Substantial increase in burden

Slight increase in burden

Moderate increase in burden

No increase in burden

9. Have you received any complaints from patients (or their families) due to the supply instability of valproate preparations?

9-1. Seizure increase from medication change

Yes

No

9-2. Adverse effects occurred from medication change

Yes

No

9-3. Anxiety about not being able to obtain their medication

Yes

No

9-4. Anxiety about medication quality

Yes

No

9-5. Dosing errors from changes in medication appearance

Yes

No

9-6. Difficulty swallowing due to change in form (tablets, powders, liquids)

Yes

No

9-7. Inability to use the public assistance medical system due to pharmacy change

Yes

No

9-8. Other (open-ended)

10. Have you held conferences or established protocols with doctors regarding patient restrictions or prescription changes due to the supply instability of valproate preparations (excluding individual prescription inquiries)?

Conducted conferences or exchanges of opinions

Making medication changes without the clarification inquiries due to PBPM

None

11. Before the situation of generic drug supply instability, what was your approach to changing to generics? Please respond based on how you handled other medications besides antiepileptic drugs.

11-1. Did you recommend changing to generics for patients whose seizures were controlled with brand-name drugs?

Actively recommended

Recommended

Did not recommend

Recommended not to change

11-2. Did you recommend changing to generics for newly started antiepileptic drug patients?

Actively recommended

Recommended

Did not recommend

Recommended not to change

11-3. Did you recommend changing to generics for patients whose seizures were not controlled with brand-name drugs?

Actively recommended

Recommended

Did not recommend

Recommended not to change

12. In the current situation of generic drug supply instability, what is your approach to changing to generics? Please respond based on how you handle other medications besides antiepileptic drugs.

12-1. Do you recommend changing to generics for patients whose seizures are controlled with brand-name drugs?

Actively recommend

Recommend

Do not recommend

Recommend not to change

12-2. Do you recommend changing to generics for newly started antiepileptic drug patients?

Actively recommend

Recommend

Do not recommend

Recommend not to change

12-3. Do you recommend changing to generics for patients whose seizures are not controlled with brand-name drugs?

Actively recommend

Recommend

Do not recommend

Recommend not to change

13. Among the methods of reporting changes to medical institutions when dispensing antiepileptic drugs, including valproate preparations, to generics, which one do you use most frequently?

FAX

Telephone

Noted in patient's medicine notebook

Other (open-ended)

14. Please provide any other comments or opinions.
